# Comparison of Intraosseous and Conventional Dental Anesthesia in Children—A Scoping Review

**DOI:** 10.3390/dj13070326

**Published:** 2025-07-18

**Authors:** Anastasia Dermata, Sotiria Davidopoulou, Aristidis Arhakis, Nikolaos Dabarakis, Konstantinos N. Arapostathis, Sotirios Kalfas

**Affiliations:** 1 Pediatric Dentistry Department, Aristotle University of Thessaloniki, 54124 Thessaloniki, Greece; adermat@dent.auth.gr (A.D.); arhakis@dent.auth.gr (A.A.); koarap@dent.auth.gr (K.N.A.); 2 Operative Dentistry Department, Aristotle University of Thessaloniki, 54124 Thessaloniki, Greece; sdavidop@dent.auth.gr; 3 Dentoalveolar and Implant Surgery Department, Aristotle University of Thessaloniki, 54124 Thessaloniki, Greece; nikosd@dent.auth.gr; 4 Preventive Dentistry, Periodontology and Implant Biology Department, Aristotle University of Thessaloniki, 54124 Thessaloniki, Greece

**Keywords:** intraosseous anesthesia, intraosseous anesthesia, dental anesthesia, children

## Abstract

**Background/Objectives**: The main purpose of the present scoping review was to map and explore the efficacy of computer-controlled intraosseous anesthesia (CCIA) in comparison with conventional dental anesthesia in pediatric dental patients. Secondarily, this study aimed to compare the acceptance and preference factors between CCIA and conventional dental anesthesia in children. Given the limited and heterogeneous nature of the available literature, this review aimed to identify gaps and scope the extent of research conducted in this area, providing a foundation for future, more targeted studies. **Methods**: The search was conducted in 19 electronic databases, and the appropriate studies were identified according to PRISMA-ScR guidelines. Only split-mouth randomized controlled clinical trials that reported on the clinical outcomes of CCIA in children were included. Two reviewers worked independently on the screening and selection of the studies. The same two reviewers carried out the data extraction and the risk of bias assessment, using the Cochrane risk of bias tool. Due to the exploratory nature, this review focused on mapping the characteristics, outcomes, and research trends rather than synthesizing effect sizes. **Results**: Out of 841 papers, 2 randomized clinical trials were ultimately included in the scoping review. The outcomes were categorized as primary (including results that answered the focus question) and secondary (relating to additional quality characteristics). Regarding the primary outcomes, in both studies, intraosseous anesthesia was efficacious in achieving the adequate level of anesthesia. One of the secondary outcomes was the acceptance and preference of CCIA in comparison with conventional dental anesthesia in children. The limited number and the high risk of bias in existing studies highlight the necessity for more comprehensive and high-quality research. **Conclusions**: The selected studies support the assertion that CCIA is a promising technique since it results in less pain perception and is preferred by patients compared to conventional local anesthesia. However, the existing literature is limited and at high risk of bias. Thus, further targeted investigations are needed to evaluate and yield more definitive results regarding the superiority of CCIA.

## 1. Introduction

Providing dental treatment with the least possible discomfort has always been highly important and necessary for an overall positive dental experience. Dental anxiety affects approximately 10–20% of children globally, with some studies reporting rates as high as 30% depending on age and cultural context [1]. Pain control during dental procedures is commonly achieved by local anesthesia (LA) using injection. While this method successfully controls pain throughout the procedure, the anxiety and negative response produced prior or during the administration of the anesthetic solution remains an issue for patients, especially children. The administration of LA is regarded as one of the most painful dental procedures and, furthermore, is related to dental anxiety and fear [1,2].

The pain related to LA is a result of soft tissue trauma during needle insertion along with tissue tension during the infusion of the anesthetic solution [3]. Considering the pain and discomfort, a proper anesthesia technique is of the utmost importance, particularly for younger patients for whom cooperation is an important challenge.

Periapical LA and inferior alveolar nerve block anesthesia are the most usual types of LA in pediatric dentistry, in which the infiltration of the anesthetic solution interrupts the impulse transmission through the injection area, resulting in the loss of the sensory perception and producing a deep anesthetic effect that permits a wide range of treatments from simple to complex ones. Though generally efficacious, these delivery methods are sometimes regarded as painful and are related to self-induced soft tissue injury. Furthermore, the efficacy of conventional anesthesia can be questionable, especially in cases of acute pulpitis.

Intraosseous anesthesia is the injection of the anesthetic solution directly into the cancellous bone after cortical bone penetration. The cancellous bone structure allows the fast diffusion of the anesthetic agent and, as a consequence, the immediate onset of anesthetic effect. Computer-controlled delivery systems of anesthetics were introduced in order to produce less pain during the anesthesia by controlling the pressure as well as the speed of the injected solution. Computer-controlled LA systems deliver the anesthetic solution at a slow and stable rate, pressure, and volume, providing less discomfort during injection. In this context, computer-controlled intraosseous anesthesia (CCIA) was developed to decrease a patient’s discomfort while increasing efficacy through the injection of the anesthetic solution into the cancellous bone in a direct way. By bypassing soft tissue and cortical bone barriers, this technique enables rapid and profound anesthesia with a more localized effect. As a result, the anesthesia is considered both fast and effective [4]. CCIA can provide a good anesthetic effect for one or more teeth within a quadrant. The anesthetic solution is delivered near the root, anesthetizing the tooth to be treated as well as its mesial and distal counterparts with a single penetration. CCIA systems can be used as either the primary method or as a supplement to the conventional regional anesthesia method [4,5].

Along with the efficacy of the anesthesia, the acceptance of the intraosseous technique, as reported by the patients, together with the preference over the other available methods, are important factors to consider, as any improvement in regarding painless and acceptable procedures is not to be neglected, especially as far as the dental treatment of children is concerned [6,7].

In recent years, significant emphasis has been placed on patient-centered approaches in pediatric dentistry. Dental care, especially for pediatric patients, involves not only the actual dental treatment but also the psychological and emotional support of children who may associate dental visits with fear or trauma. Minimalizing pain and anxiety and achieving a child’s cooperation is regarded almost as critical as the treatment itself [6]. With this in mind, alternative techniques, such as CCIA, aim to improve the actual efficacy as well as to create a positive and less intimidating experience.

Despite its many advantages, the application of CCIA anesthesia in pediatric patients requires the careful consideration of anatomical factors, appropriate case selection, and clinician expertise. Concerns such as the proximity of the anesthesia’s application to developing tooth structures and patient cooperation during administration must be addressed through proper training and technique precision [4].

Current evidence on the use of CCIA in pediatric dental patients remains limited and scarce and there is no comprehensive review that has yet tried to approach the existing literature on this topic. Therefore, the aim of this scoping review is to provide an overview of current research related to CCIA in pediatric dentistry. Specifically, this review attempts to explore the reported efficacy and acceptance of and preference for CCIA in children compared to conventional LA, as well as to identify knowledge gaps and areas for future research.

## 2. Materials and Methods

The scoping review was carried out between 10 June and 4 November 2024, according to PRISMA-ScR guidelines. The main aim was to map and explore the existing literature on the efficacy and acceptance of and preference for CCIA compared to conventional dental anesthesia in children. The main clinical variable evaluated in the eligible studies was the efficacy of the anesthetic techniques in achieving adequate anesthesia. The criteria for evaluating efficacy were the need for additional anesthesia and patient-reported pain during treatment. Emphasis was placed on the characteristics, scope, and gaps within the available studies.

This protocol was registered with the Open Science Framework (registration DOI: https://doi.org/10.17605/OSF.IO/NZPVW; registration date: 15 April 2025) and includes a detailed outline of the research question, inclusion and exclusion criteria, planned search strategy, data extraction methods, and risk of bias assessment tools.

**Figure 1:**
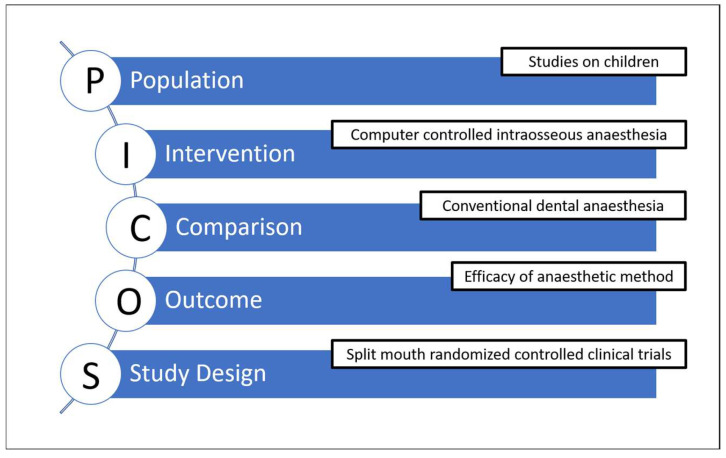
The PICOS framework for inclusion and exclusion criteria.

The primary aim was to explore and map the existing evidence regarding whether CCIA offers efficacy and other advantages over conventional LA in children, rather than to definitively establish superiority. Accordingly, the focus question was about the extent, characteristics, and outcomes reported in the current literature, recognizing that the limited number of high-quality comparative studies precludes quantitative synthesis. The inclusion and exclusion criteria according to PICOS framework are shown in Figure 1.

- Population: Studies on children. (Animal or experimental studies and studies on adults were excluded.)
- Intervention: Computer-controlled intraosseous dental anesthesia.
- Comparison: Conventional dental anesthesia.
- Outcome: Studies reporting dental anesthesia efficacy.
- Study design: Only split-mouth randomized controlled clinical trials were included.

Two authors (A.D. and S.D.) independently searched 19 electronic databases in June 2024. The main search strategy consisted of the terms “(anaesthesia, anesthesia, intraosseous, dental anaesthesia, local anesthesia, children)”. No limits were set as regards to demographic characteristics of the trial population, language, or publication date. Other study types aiming to describe or explore outcomes relevant to CCIA use were also considered for mapping.

Database and register selection was conducted with the intention of covering of relevant content, which was also based on quality and accessibility (PubMed, Science Direct, Cochrane Reviews, Cochrane Protocols, Cochrane Trials, Scopus, Ovid, BMJ Evidence-Based Medicine, Web of Science, ProQuest, Livivo, ClinicalTrials.gov, metaRegister of Controlled Trials). Gray literature databases were also included for the coverage unpublished or non-indexed evidence (Google Scholar, greylit.org).

The following database search strategy was used: PubMed (“anaesthesia”) OR (“anesthesia” [MeSH Terms]) OR (“anesthesia” [All fields]) OR (“anesthesias” [All fields]) AND (“intraosseous”). Science Direct: TITLE-ABS-KEY: (“Intraosseous”) AND (“anaesthesia”). Google Scholar: ALL IN TITLE: (“Intraosseous Anaesthesia”). Cochrane Reviews: (“Intraosseous Anaesthesia”). Cochrane Protocols: (“Intraosseous Anaesthesia”) Cochrane Trials (“Intraosseous Anaesthesia”). Scopus: TITLE-ABS-KEY (“intraosseous”) AND (“anaesthesia”) OR (“anesthesia”). Ovid: ALL IN TITLE: (“Intraosseous Anaesthesia”). BMJ evidence-based medicine: TITLE-ABS-KEY (“intraosseous”) AND (“anaesthesia”) OR (“anesthesia”). Web of Science™: (“intraosseous”) AND (“anesthesia”) OR (“anaesthesia”). ProQuest Noft: (“Intraosseous Anaesthesia”). greylit.org: (“Intraosseous Anaesthesia”). Ethos: (“Intraosseous Anaesthesia”). Livivo: (“Intraosseous Anaesthesia”) ClinicalTrials.gov: (“Intraosseous Anaesthesia”). metaRegister of Controlled Trials: (“Intraosseous Anaesthesia”).

The transparency and reproducibility of the review process was ensured through developing and testing using a standardized data extraction method. For the studies included, basic elements were recorded, such as authorship, year of publication, country, study design, sample size, patients age, anesthesia methods used, outcome measures, and results related to efficacy, elements that evaluate patient acceptance and patient preference. Missing data were noted. All extracted data were verified by both reviewers independently to minimize errors and subjective interpretation. The scarcity of eligible studies is a result of a gap in the existing literature rather than a limitation of the search strategy.

The selection was based firstly on the title, then on the abstract, and finally, on the full text. Trials meeting the inclusion criteria or trials missing information underwent final full-text evaluation. The two authors followed a standardized protocol to ensure the consistent application of inclusion and exclusion criteria during title, abstract and full-text screening stages. Authors ensured familiarity with the review protocol, then acquainted themselves with review objectives, inclusion, exclusion criteria, and PICOS framework. Screening was conducted using the Rayyan platform. Studies were included if they aligned with the broad mapping aim. Studies that lacked sufficient information or did not meet criteria during the full-text review were recorded with reasons for exclusion provided. The risk of bias was assessed but was not a criterion for inclusion as the focus was on describing the breadth of existing research. If a study was judged as not eligible, the reason for the exclusion was noted. In case of discrepant evaluations, the authors re-examined and discussed the study to reach an agreement. No adjudication by a third reviewer was needed. Reasons for the exclusion of a study following full text screening were reported. As the objective was mapping, data synthesis was descriptive rather than quantitative, and no meta-analysis was performed due to heterogeneity and limited data.

## 3. Results

### 3.1. Study Selection

**Figure 2:**
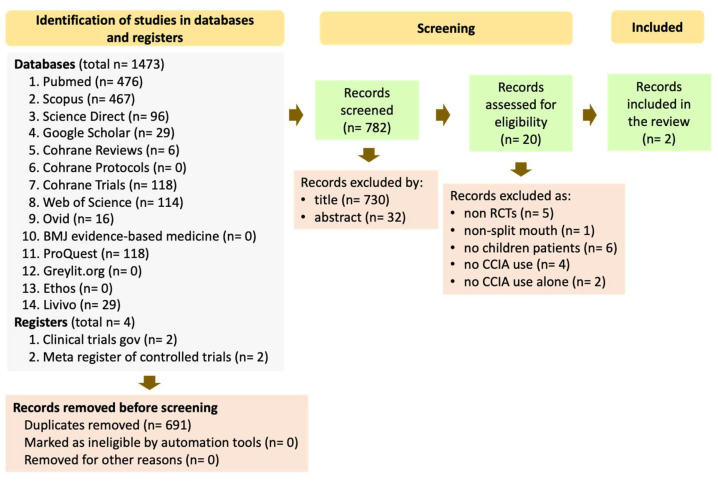
Flowchart for study selection according to PRISMA-ScR guidelines.

The review of 14 electronic databases and 2 registers yielded 1473 and 4 papers, respectively (Figure 2). After duplicate removal, 782 publications remained. The four papers in the registers were removed as duplicates because they existed as published articles in the database records. The remaining publications were screened by title and by abstract, leading to a selection of 52 and 20 papers, respectively. Finally, the 20 articles were assessed on their full texts. Among them, 17 were excluded for the following reasons: (a) non-randomized controlled trials, (b) no split-mouth design, (c) trials with adults, (d) no use of CCIA, and (e) supplementary use of CCIA. In the end, only two studies fulfilled the broad inclusion criteria for this mapping review, underscoring the need for more comprehensive, high-quality research to establish firm clinical conclusions (Figure 2).

The outcomes of the review were categorized as primary, i.e., they answered the focus question, and secondary, i.e., they possessed additional quality characteristics of the included studies.

### 3.2. Primary Outcomes

The main clinical variable evaluated in the eligible studies was the efficacy of the anesthetic techniques for achieving adequate levels of anesthesia. The evaluation criteria for efficacy were (a) the need for additional anesthesia and (b) the pain reported by the patient during treatment.

### 3.3. Secondary Outcomes

Other outcomes reported in the included studies were (a) pain perception during the application of anesthesia, (b) disruptive behavior during treatment, and (c) patient-preferred method.

### 3.4. Features of Included Papers

The characteristics of the selected studies are listed in Table 1. The first study was a randomized controlled clinical trial conducted using a split-mouth design. The second study had a mixed parallel and split-mouth design, though only the results of the split-mouth sample were included in the present review. The sample sizes was 30 and 50 for the first and the second studies, respectively. The mean ages of the patients were 9.0 ± 2.3 years for the first and 7.6 ± 2.0 years for the second study.

The anesthesia protocol included the use of topical anesthetic before local anesthesia was administered. The topical anesthetic used was xylocaine 2% for the first and benzocaine 20% for the second study, applied for 1 to 2 min and for 1 min, respectively. The anesthetic solution used was 4% articaine with adrenalin at a 1:200,000 ratio in the first study and 2% lidocaine with epinephrine at a 1:100,000 ratio in the second study. As far as the anesthesia site was concerned, the first study carried out an infiltrative technique for the maxilla and an inferior alveolar nerve block for the mandible during conventional anesthesia while the second study used the inferior alveolar nerve block and compared it with the computer-controlled intraosseous anesthesia technique. Only permanent first molars were included in the first study, and only mandibular primary molars were included in the second study.

The primary outcome for the first study was the pain experienced during the anesthesia that was assessed using the VAS. In the second study, the assessment of the pain was carried out with the application of the Wong–Baker visual pain scale, while the disruptive behavior was analyzed using the FLACC scale [7,8].

**Table 1:** Characteristics of the two included studies.

Study Characteristics	Smail Faugeron et al., 2019 [9]	Prol Castelo et al., 2022 [10]
**Design**	Split mouth, RCT	Split mouth, RCT
**Age**	9.00 ± 2.3	7.6 ± 2.0
**Sample (No. of participants)**	30	50
**Intervention**	CCIA vs. conventional LA in contralateral first permanent molars.	CCIA, vs. inferior alveolar block anesthesia (BA) in contralateral deciduous molars.
**Outcome criteria**	Pain of insertion, pain during anaesthesia latency need for reinforcement, pain during treatment.	Pain, physical reaction, need for reinforcement, overall behavior, post-operative morbidity, preference.
**Results**	CCIA: Statistically insignificant less pain upon interaction and treatment, shorter latency.	Statistically significant differences in favor of CCIA: less injection pain, less post-operative morbidity, less post-operative complications, patient preference.
**Conclusions**	Reduced pain associated with CCIA.	CCIA is more efficacious for invasive procedures and more advantageous in terms of acceptance and preference, compared with inferior alveolar BA.
**Limitations stated by the authors**	Operators not blinded to randomization due to different devices used.	Limitations inherent to the inclusion criteria: age, cooperation, teeth involved, dental procedure. No assess of the anesthetic effects and duration, no specific tools for determining the efficacy of the system.

### 3.5. Risk of Bias

The risk of bias of the selected studies was evaluated by the two reviewers (A.D. and S.D.) with the use of the revised Cochrane risk of bias assessment tool for randomized trials (Rob2) [11]. This tool was used to evaluate five different domains: the randomization process (random sequence generation and allocation concealment), deviation from planned interventions (blinding of participants and personnel, adherence to intervention), missing outcome data, the measurement of the results (blinding of outcome assessment), and the selection of mentioned results (selective reporting of outcomes). Each of these domains was evaluated as having a low risk, a high risk, or an unclear risk of bias (Table 2).

With regard to the exploratory and mapping nature of this scoping review, the risk of bias assessment was conducted primarily to characterize the quality and heterogeneity of the evidence base rather than to exclude studies. To achieve objectivity and consistency, the two reviewers (A.D. and S.D.) independently assessed each included study domain with the use of the RoB 2 tool. Any discrepancies were resolved through discussion and consensus. No third-party adjudication was required. A calibration exercise took place prior to the formal assessment, during which the reviewers applied the RoB 2 tool to a sample study not included in the final review, with the intention of aligning their interpretations of the criteria.

## 4. Discussion

Pain control in dental care is of paramount importance, especially when treating children. Despite limiting pain during treatment, the administration of LA is associated with discomfort, anxiety, and pain. LA-associated pain perception is a result of two distinct causes. The first is the needle penetration that triggers a brief, intense pain. The second relates to the activation of nociceptors by the compounds of the anesthetic solution and the tension created in the soft tissue by the injection. The latter sensation is regarded as more intense and more prolonged [12].

A periapical injection and inferior alveolar nerve block are commonly used techniques for applying LA. New delivery methods for LA aim to offer efficacious anesthesia while being comfortable for the patient. Among them, CCIA is presented as an alternative to conventional LA, allowing the injection of the anesthetic solution directly into the cancellous bone around the tooth that is intended to be anesthetized [11,13]. The anesthetic compound quickly diffuses in the bone and exerts an almost immediate effect. Careful needle insertion and slow injection can possibly result in less discomfort for conventional LA. Yet, CCIA enables a more certain and predictable result [9,14,15].

The efficacy of LA is indisputable for successful dental treatment; however, its acceptance is a critical factor too. The level of acceptance is determined by whether every single stage before, during, and after LA application considered manageable by the patient, without causing any major discomfort [6,16]. Elements such as pain, tension, fear, bad taste, and numbness are to be considered when assessing acceptance. The preference for certain methods factor is equally of paramount importance when aiming to create a pleasant dental experience [6].

An important consideration in the pediatric population is a child’s cooperation during the administration of local anesthesia. Pediatric patient response can be influenced not only by the possible pain during local anesthesia application but also by their dental-related anxiety [6]. Alternative local anesthesia methods such as CCIA, which involves faster onset and potentially reduces discomfort, could possibly result in improved cooperation and compliance during dental treatment. Furthermore, a positive experience during the injection of anesthetic using CCIA could influence a child’s long-term cooperation and attitude with regard to dental treatment, which may result in less dental anxiety and avoidance in adolescence and adulthood [4].

This scoping review aimed to explore existing evidence on CCIA in children. Only randomized controlled trials (RCTs) were considered, focusing specifically on split-mouth studies to minimize inter-individual variability and improve the reliability of findings [17]. Due to these inclusion criteria, only two papers were ultimately included. Upon extending the present literature search to include other studies (with a non-split-mouth design), no other high-quality RCTs was identified. The double, split-mouth, and parallel-arm design study of Smail-Faugeron et al. compared the pain caused by conventional LA and CCIA in children [9]. Prol Castelo et al. [10] also evaluated CCIA in comparison with the conventional method in a split-mouth randomized controlled trial. The former study found no significant differences between application methods. The assessment of the efficacy involved the need for additional anesthesia and the pain reported by the patients during dental treatment. The pain experienced throughout the dental treatment was evaluated with a visual analog scale (VAS) [8]. Mean VAS scores were 1.07 ± 1.76 cm for the intraosseous method and 0.53 ± 0.82 cm for conventional anesthesia. Children reported less pain during dental treatment with the conventional method than with the intraosseous technique, but the difference was not statistically significant. In the latter study, efficacy was assessed via the pain that children experienced during dental treatment and reported with the use of the Wong–Baker scale [18]. Children’s overall cooperation during dental procedures was also used to indirectly evaluate possible discomfort, using the Frankl scale [19]. Again, there were no statistically significant differences between the two methods.

Statistically insignificant was also the difference in pain felt upon needle insertion and the injection of the anesthetic, although CCIA appeared to be less painful [14]. Mean VAS scores were 0.73 ± 1.31 cm for CCIA and 1.43 ± 1.45 cm for conventional LA. The mean (95% CI) for the difference in paired proportions was −0.70 ± 0.36 cm (−1.44 to 0.04). The second study [18] showed CCIA to be significantly less painful during injection (*p* = 0.005) and to exhibit lower postoperative morbidity (*p* = 0.009). Nevertheless, the computer-controlled intraosseous technique was compared only with the inferior alveolar nerve block, which is often related to more pain than conventional periapical anesthesia [18]. In terms of acceptance of the method, these results favor CCIA since any improvement for painless anesthesia is important as far as the patient is concerned. This aspect is further supported by the fact that the majority (76%) of the patients expressed their preference for CCIA rather than IANB [18].

As mentioned previously, only two studies were included in this scoping review. Only one of them provided results that demonstrated the significant superiority of CCIA. Both studies were evaluated to have a high overall risk of bias, with the risk of overestimating any benefits of CCIA.

The risk of bias in the two studies can be mainly attributed to lack of blinding and issues in participant selection. The lack of blinding may have influenced both self-reported and operators reported pain scores and thus may have overestimated any benefits of CCIA. Moreover, the clinical relevance of these benefits is not certain as clinically important differences regarding pain scores in pediatric dental procedures are not well established [1].

The review results outline the need for more standardized RCTs, aiming to address the current limitations and, furthermore, provide a clearer assessment of the overall reliability of the current evidence base. Issues such as sample size, consistent pain measurement, and tooth type are important to consider and should be standardized in future research. This scoping review could be considered a tool for mapping emerging or scarce evidence, helping to identify research gaps and informing future studies on pediatric dental anesthesia. This approach provides a foundation for conducting more clinical trials and rigorous systematic reviews in the future.

Clearly, the present data are limited, and any comparison is possibly based on inadequate scientific evidence. More studies are needed to assess CCIA performance and advantages and to demonstrate whether it should be recommended as a better alternative to other LA methods during dental treatment.

## 5. Conclusions

Based on the results of this scoping review, it can be assumed that CCIA is a promising technique. According to the selected studies, it is related to less pain perception compared to conventional LA in children. One selected study supports the superiority of CCIA in terms of patient preference. However, the existing literature is limited and has a high risk of bias. Thus, more studies are needed to evaluate the superiority of CCIA.

## Figures and Tables

**Table 2:** Evaluation of the risk of bias for the two included studies.

Bias Domain	Smaïl-Faugeron et al., 2019 [9]	Prol Castelo et al., 2022 [10]
**Bias arising from the randomization process**	low	low
**Bias due to deviation from intended interventions**	high	high
**Bias due to missing outcome data**	low	low
**Bias in the measurement of the outcome**	high	high
**Bias in the selection of the reported results**	low	low
**Overall risk of bias**	high	high

## Data Availability

All referenced data is available through the original sources cited within the review. Due to the nature of the study, no data are available for public access, and there are no privacy or ethical concerns regarding data sharing.
